# STRIVE PNG: using a partnership-based approach in implementation research to strengthen surveillance and health systems in Papua New Guinea

**DOI:** 10.1186/s12961-022-00840-3

**Published:** 2022-04-02

**Authors:** Rachael Farquhar, Annie Dori, Sarah MacCana, Nakapi Tefuarani, Evelyn Lavu, Alyssa Barry, Stephan Karl, Leo Makita, Leanne Robinson, Moses Laman

**Affiliations:** 1 Burnet Institute, Melbourne, Australia; 2 PATH, Port Moresby, Papua New Guinea; 3 Partnership Brokers Association, Wellington, New Zealand; 4 University of Papua New Guinea School of Medicine and Health Sciences, Port Moresby, Papua New Guinea; 5 Central Public Health Laboratory NDoH, School of Medicine and Health Sciences, PNG University of Papua New Guinea, Port Moresby, Papua New Guinea; 6 Deakin University, Geelong, Australia; 7 James Cook University, Smithfield, Australia; 8 Papua New Guinea Institute of Medical Research, Madang, Papua New Guinea; 9 National Malaria Control Program, National Department of Health, Port Moresby, Papua New Guinea; 10 Walter and Eliza Hall Institute, Parkville, Australia

**Keywords:** Localization, Partnership, Partnership brokering, Implementation research, Papua New Guinea, Vector-borne disease, Sustainability, Translational research

## Abstract

Successful implementation research requires effective and equitable relationships between policy-makers, researchers and implementers to effect evidence-based systems change. However, mainstream research grant models between Global North and Global South institutions often (unintentionally) reinforce power imbalances between partners, which result in missed opportunities for knowledge and learning exchange between policy-makers, researchers and implementers.

This case study, centred on the STRIVE PNG project, describes how a partnership-based approach has been used to establish, maintain and review effective and equitable relationships between 13 partner organizations (independent research institutes, government health agencies and public health laboratories) to strengthen surveillance and health systems in Papua New Guinea (PNG). We provide an overview of key terms (with supporting conceptual frameworks), describe selected partnership processes and tools used within the project, and share observations regarding early outcomes achieved through this approach.

## Main text

### Why: the case for partnering in implementation research

Successful implementation research requires effective and equitable relationships between policy-makers, researchers and implementers to effect evidence-based systems change. However, mainstream research grant models often (unintentionally) reinforce donor–recipient relationships between well-resourced research organizations from high-income countries in the Global North, and “local” research organizations and government ministries from low- to middle-income countries in the Global South [1, 2]. Limitations include limited (or non-meaningful) engagement of local researchers or policy-makers in research design or proposal writing; authorship of publications led or dominated by Global North researchers; or lack of understanding of political implications of sharing research data and findings [3]. These donor–recipient relationships lead to missed opportunities for knowledge and learning exchange between sectors (policy-makers, researchers and implementers), resulting in research that remains academic, policy that is not based on evidence, or service delivery that fails to learn from past experience [4].

There is growing recognition that establishing equitable relationships, or partnerships, between key stakeholders involved in translational research can overcome these limitations [5]. This case study, centred on the STRIVE PNG project, describes how a partnership-based approach has been used to establish, maintain and review effective and equitable relationships among 13 partner organizations (independent research institutes, government health agencies, and public health laboratories) to strengthen surveillance and health systems in Papua New Guinea (PNG). We begin with an overview of key terms (with supporting conceptual frameworks), before describing selected processes and tools used within the project, and share observations regarding early outcomes achieved through this approach.

### A note on terminology: “partnership”, “brokering” and the partnering cycle

**Fig. 1:**
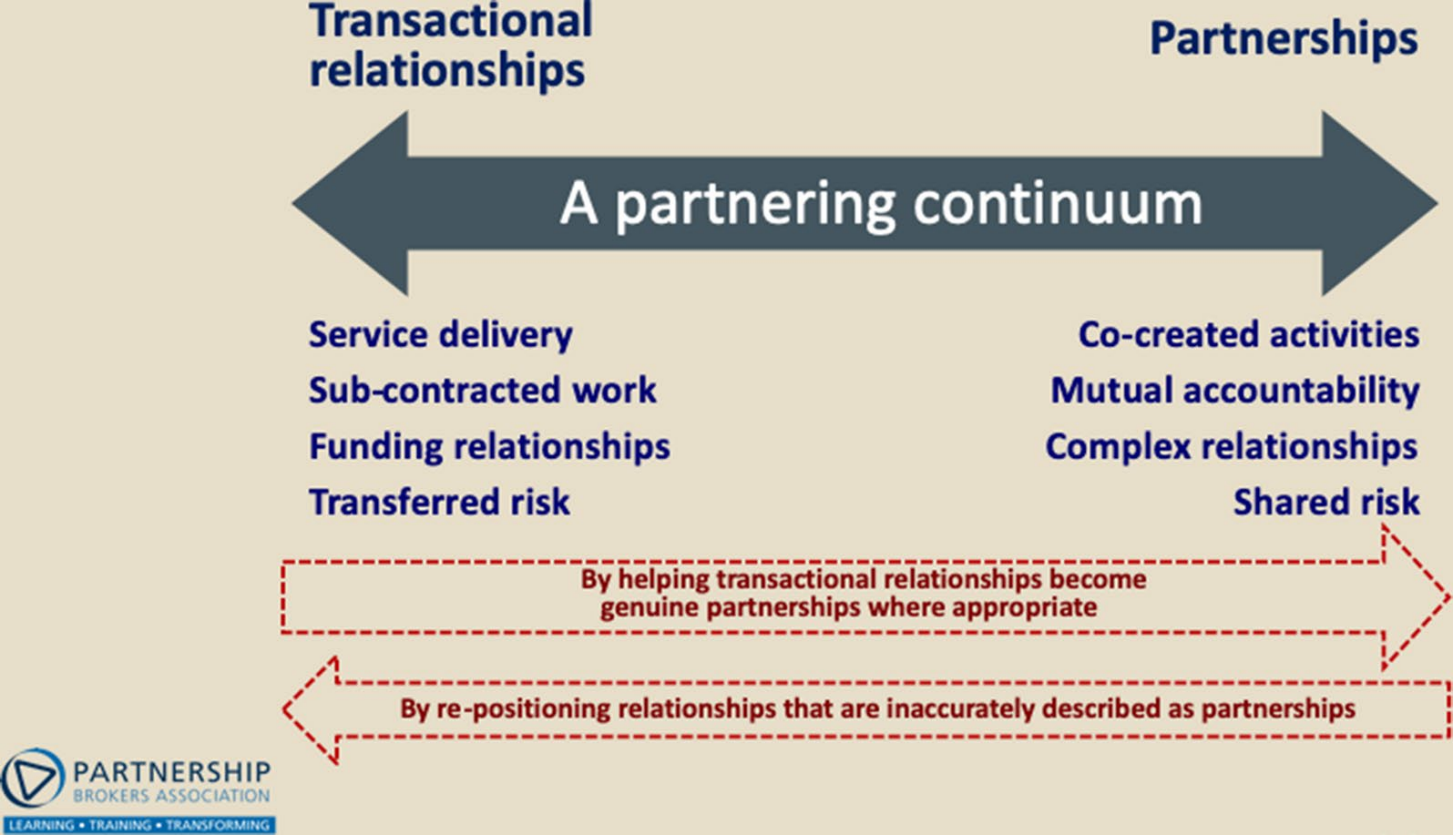
Partnering continuum

The term “partnership” is used widely to describe significantly different relationships and arrangements [6]. In the absence of a universally agreed definition, STRIVE PNG has adopted a typology proposed by the Partnership Brokers Association (PBA),^1^ who use the partnering continuum (Fig. 1 below) to help potential partners collectively agree where their relationship sits along a scale of “transactional relationships” to “collaborative partnerships”.

It is important to note that the continuum is a conceptual tool to help partners agree on “fit-for-purpose engagement”, and does not reflect a judgement of “good/bad”. This is because the type of relationship that is appropriate for a specific group of organizations will vary according to what the group is trying to achieve. For example, a contract-based relationship is appropriate when one organization is purchasing goods and services from others. However, if the group is trying to effect transformational, systems change, they will likely need a more collaborative relationship, with joint decision-making and shared risk.

In using the continuum, groups are also reminded that relationships are not static and that partners may agree over time that they need to shift their relationship in one direction (or the other) depending on their partnership’s evolving aims (becoming more ambitious, or less), changes in their operating environment (responding to opportunities or barriers), or even internal changes (less or more time, less or more permission space for risk, etc.).

“Partnership brokering” describes “the essential intermediary function that enables partners to work together well (equitably) and ensure the maximum effectiveness of their partnership” [7]. Successful partnerships rest on one (or more) person who has taken on these functions, often intuitively and unofficially [7]. With the formation of the Partnership Brokers Accreditation Scheme (PBAS) in 2003 and its provision of a vocational qualification pathway for those working in multi-stakeholder collaborations, there is increasing recognition of the specialized role of partnership brokers—those with the skills, experience and mindset required to work “on”, not just “in”, the partnership. Brokers help partners “walk the talk” of their shared vision, by providing skilled process management across all phases of the partnering cycle (see below). Brokers may be internal (managers within a partner organization) or external (independent professionals contracted by one of the partners) [7].

**Fig. 2:**
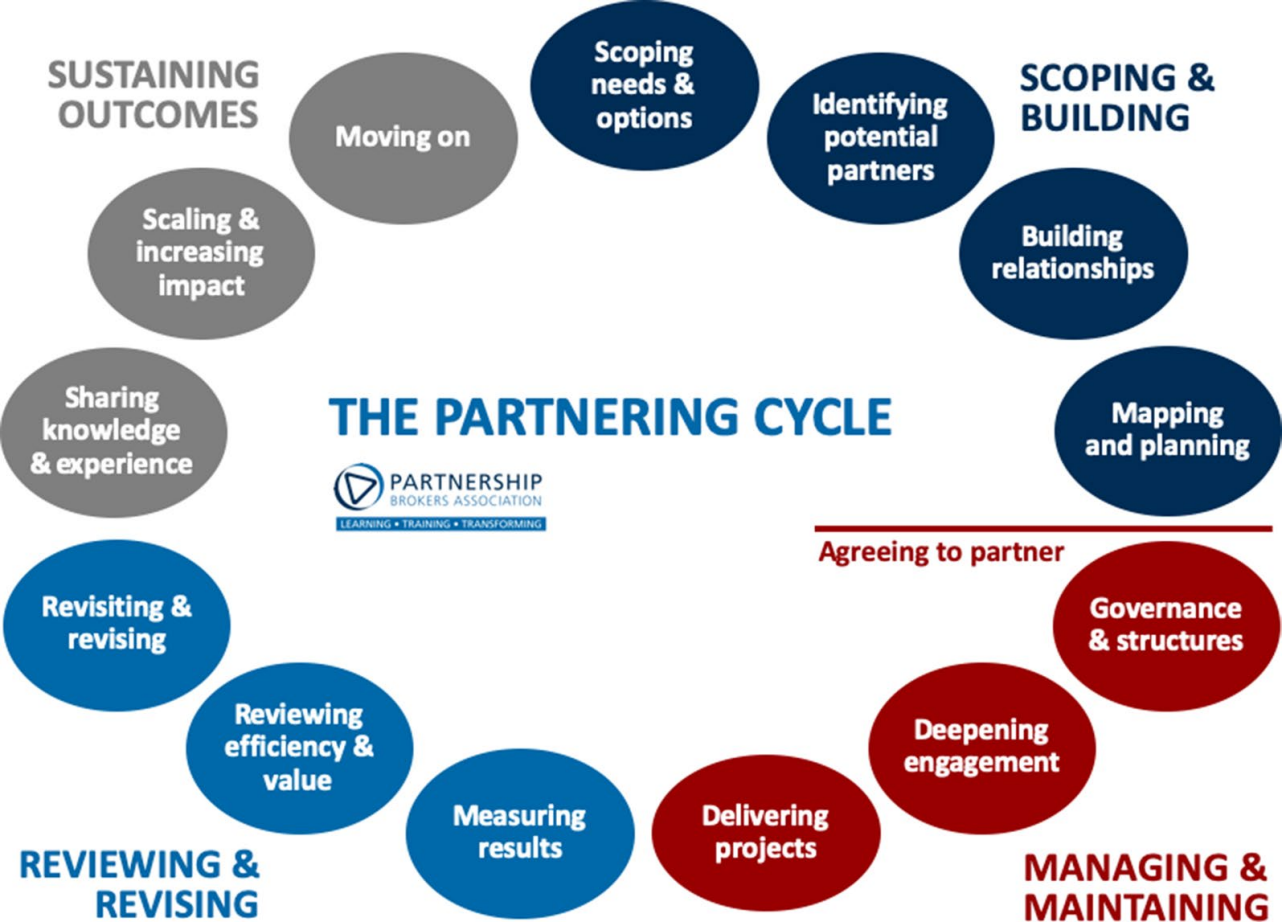
Partnering cycle

The “partnering cycle” (Fig. 2) describes key phases associated with building, managing, reviewing and sustaining a partnership. Brokers use key processes and tools at key points in the cycle to ensure that the partnership’s business processes (membership, design, governance, communications, budgeting, monitoring and evaluation, etc.) strengthen equity between partner organizations and unlock the partnership’s maximum value. In reality, the cycle is dynamic, and partners will often work across multiple phases simultaneously, or backward and forward as needed. The following case study uses the partnering cycle to look at key processes and tools used in selected stages of the cycle, and shares observations on early outcomes achieved through the use of these processes and tools.

### Background to STRIVE PNG

STRIVE PNG, an abbreviation for “stronger surveillance and systems support for rapidly identifying and containing resurgent or resistant vector-borne pathogens in PNG”, is a 4-year (2019–2022) project funded by the Australian Department of Foreign Affairs and Trade's (DFAT) Indo-Pacific Centre for Health Security—Stronger Systems for Health Security programme. STRIVE PNG collaborates with partners from 13 leading research institutes, health departments and public health laboratories in PNG and Australia to:

1. jointly strengthen and enhance surveillance and health system capacity by developing an innovative approach to surveillance and response, which are appropriate and sustainable to PNG; and
2. develop comprehensive and effective partnerships between implementation and research organizations that can realize meaningful and sustained change.

The STRIVE PNG project was first initiated by several key partners who shared a vision of enhancing health security through the Pacific region and had been working together since January 2016 on an international cooperation project, the Australia-China-PNG Trilateral Malaria Project. Through exposure to the partnership-based model used in the Trilateral Malaria Project, which involved shared decision-making, a principles-based approach and mutual accountability [8], health research partners in Australia and PNG determined that this way of working (in contrast to more traditional research grant models) fostered trust and genuine ownership and strengthened impacts across PNG organizations and, importantly, the PNG health system. In this way, STRIVE PNG represents an example of transferring good practice lessons from the international development sector [9–11] to implement effective translational research in an implementation setting [12].

*STRIVE is different in a sense that the partnership is the core part of the project. Often you have a research project and build the partnership to implement the project’s activities. STRIVE is the other way around.* —STRIVE PNG Programme Director

## STRIVE PNG’s Partnership Management Unit

STRIVE PNG’s Australian and Papua New Guinean Co-Principal Investigators had been involved in multi-institutional research grants for > 15 years and recognized that working in partnership required specific skill sets and resourcing. At inception, investigators decided to supplement the policy and technical roles of partners with a dedicated Papua New Guinean “project partnership manager” to provide both project management and internal partnership broker functions: “*We wanted to recruit someone neutral, someone that does not belong to any particular organization, and embodies partnership principles and mindset*” (STRIVE Co-Principal Investigator). At the 1-year mark, STRIVE PNG’s Australian-based internal project manager’s role was also expanded to include specific internal partnership brokering responsibilities. STRIVE PNG’s two internal brokers are supported by an external and PBA-accredited partnership broker, who provides partnership facilitation, design, mentoring and coaching support to the internal brokers and broader partners. This Partnership Management Unit is now also called on to provide brokering support to the PNG Health Department’s National Malaria Control Programme in its relationships with other stakeholders, including PNG government agencies, international donors, multilateral organizations and nongovernmental organizations.

In the following sections, we share three examples which describe the process (methodology) and tools (exemplars, templates) used by STRIVE PNG at selected key stages within the partnering cycle. In each example, we also share some early observations of the impact of these processes and tools on partner relationships, with reference to early outcomes where appropriate.

## Example 1: principles-based relationships

**Table:** 

Stage	Process/how (tool)
Building relationships	Co-creation of guiding principles and corresponding behaviours

At their first inception meeting (July 2018), STRIVE PNG’s Co-Principal Investigators invited nominated programme directors (senior personnel from Australian and PNG partner institutions) to attend a first meeting and discuss both the “what” (technical work-planning) and “how” (principles-based relationships) to guide their joint work. This conversation on “how”, facilitated by the external partnership broker, generated a set of guiding principles and corresponding (observable) behaviours which partners agreed to abide by in their work together (Table 1 below). This was subsequently incorporated into the STRIVE PNG partnership agreement, with acknowledgement that partners would reflect on the extent to which they, and others, were practicing these principles and behaviours through internal partnership health checks.

**Table 1:** STRIVE PNG’s guiding principles and behaviours

Guiding principles	For example, this means…
Building common purpose along a shared journey	Working in ways that help partners to build a common vision and sense of purpose (why are we doing this and why are we doing this together). This includes using project planning, delivery and reporting processes to ensure partners stay connected to the partnership, and the package of work, and can share the partnership’s successes and the challenges
Promoting mutual benefit by acknowledging vulnerability as well as strengths	Partners recognize that every organization has something to give and something to receive, and it is through this two-way transfer that genuine partnership value is built
Promoting equity and voice across and within partners	Using communication and facilitation approaches which promote each partner’s confidence to give their preferences and views, and shared decision-making within the project; finding ways to give voice to the people within their teams and broader organization; finding ways to explicitly recognize and value the contributions made by each partner, especially their time invested in the partnership
Promoting ownership and sustainability within PNG institutions	Ensuring all work is undertaken based on health system and capacity needs articulated by Papua New Guinean partner organizations
Harnessing diversity across partners	Working in ways which recognize the respective skills, strengths and attributes of partners, and which are sensitive to each partner’s individual context and circumstances (e.g. planning takes account of key events in PNG’s political, administrative and health systems’ calendars, or specific financial or human resource constraints)
Promoting transparency	Demonstrating honesty, openness and frankness in communication at all levels of interaction within the partnership
Flexibility and adaptiveness	Showing ingenuity, patience and, where appropriate, a sense of humour when facing challenges within the project’s operating environment. This means remembering that we don’t have to be perfect, that what is important is to move forward—being flexible and adaptive

### Early outcomes

We see increasing recognition in the international development and research sector [13] of the important role that understanding (and harnessing) culture, relationships and identity plays in ensuring development and translational research interventions are led and owned by local actors and lead to sustainable change [14]. Papua New Guinean partnership brokers describe their work as “focusing on respect and relationships”, a way of working that resonates with Melanesian culture and the value Papua New Guineans place on reciprocity. Relationships between partners have been likened to the concept of *wantok* (family) in PNG culture:

*When a group of people are called family, we do anything for family.* —STRIVE Molecular Research Officer

*In PNG, if you want things to move, you have to belong to a tribe, a group of colleagues and a church. If you belong to these three groups, you can make things happen. For STRIVE, we have been able to set up new activities with new partners because our staff are long-term, committed members and contributors in all three of these social arenas.* —PNG Institute of Medical Research (PNGIMR) Programme Director

STRIVE PNG’s internal broker has observed that PNG’s history, involving the formation of a national identity based on unification of 800 diverse cultures with different beliefs, language and identities, has meant that Papua New Guineans have lived experience of the importance of genuine partnership and bringing people together from different places with diverse strengths and challenges [15–19]. The process of being asked to make suggestions for STRIVE PNG’s guiding principles and behaviours enabled PNG partners to draw on their personal experiences of nation-building, and apply this learning to their professional setting.

The project’s Molecular Hub is a tangible example of how partners have put STRIVE PNG’s guiding principles and behaviours into practice to achieve project results. Promoting ownership and sustainability within PNG institutions was a key driver in setting up the Molecular Hub to strengthen diagnostic capacity across three key PNG institutions (PNGIMR, National Department of Health [including Central Public Health Laboratory] and School of Medicine and Health Sciences). In order to harness diversity and foster equity, resources and technical expertise are shared across the organizations, and work plans are co-developed by the Molecular Hub team.

This way of working was shown to be of extremely high value when the Molecular Hub needed to rapidly respond to the emerging COVID-19 pandemic by establishing COVID-19 testing in March 2020. The team was able to not only scale up COVID-19 testing capacities across the PNG institutions, but also attract additional funding from the DFAT Centre for Health Security to establish new serological assays testing for recent exposure to COVID-19 to enhance the PNG government’s COVID-19 response. In doing so, the approach has also demonstrated the value-add of integrating diagnostic and surveillance approaches and expertise for multiple infectious diseases.

*Through the Molecular Hub, our team was prepared unexpectedly for COVID 19. It is a really good example of partnership, and highlighted all the strengths we had. Imagine if we didn’t have the Molecular Hub in place… *—PNG Programme Director

## Example 2: partnership agreement

**Table:** 

Stage	Process/how (tool)
Building relationships Mapping and planning Governance and structures	Partnership agreement

Organizations who are embarking on a partnership together have a range of options for documenting their engagement, including formal, legal documentation such as a contract or memorandum of understanding (MOU), as well as less formal approaches such as records of meetings or joint statements. PBA has pioneered the use of “partnership agreements” (also known as a “ways of working” document), which function as a formal, but non-legally binding, umbrella document, co-developed by all partners involved in the partnership. PBA advocates that the *process* of developing the partnership agreement is as important as the final agreement document itself [20]. Often (for legitimate reasons of expediency), one partner will draft a partnership agreement for countersigning by other partners. However, this misses a critical opportunity to build relationships and understanding between partners (and can actually slow engagement during implementation, as partners may have views or needs which were not uncovered during the agreement development process).

For STRIVE PNG, the Co-Principal Investigators emphasized that the process of developing and negotiating the partnership agreement was a way to “ensure partners feel connected, to make the project real, and to help build trust” (STRIVE PNG Programme Director). STRIVE PNG’s agreement co-development process comprised an initial face-to-face workshop between collaborators (to introduce the agreement framework and have an initial discussion on key aspects), followed by a series of one-to-one conversations between the brokering team, the Co-Principal Investigators and each partner organization. STRIVE’s brokers then synthesized the conversation outputs into a single document for simultaneous review by each partner, with additional written contributions where needed. This process was undertaken largely remotely in the “virtual space”, via phone calls, Zoom and emails. The final document was then simultaneously circulated to all collaborators, who came together in joint face-to-face (for Port Moresby-based partners) and virtual meetings to review and endorse the final document.

When confirming the signing process, the partners decided to adapt the usual endorsement process (where institutions are signatories to the document) and provide space for endorsement by each individual investigator and their institutional director. As with many research collaborations, STRIVE PNG partners had reflected that their partnership was formed on the basis of individual expertise and relationships as much as institutional capability and mandate. Endorsement at the individual level ensured that the document retained its value and authenticity, and endorsement by each organization’s director demonstrated institutional buy-in.

Table 2 sets out the framework for STRIVE PNG’s partnership agreement [20] (note: the agreement itself is not a public document, given it includes information that partners have agreed to share with each other, but not outside of the partnership).

**Table 2:** Framework for STRIVE PNG’s partnership agreement [20]

Heading	Purpose of each section
Purpose of the agreement	Ensure partners understand that the document describes the working relationships (which have been co-designed between organizations through the agreement joint drafting process) between their organizations for the duration of the project–it is non-legally binding and does not function as a contract
Background to the project and the partnership	Builds shared understanding of the project’s history and knowledge, given different partners have different levels of knowledge about the project’s genesis
The partners	Helps each partner get to know the others by providing a summary of each partner’s organizational background, given some partners were working together for the first time
Guiding principles and behaviours	Supports mutual accountability by setting out practical expectations for “how” partners will behave in the partnership
	For STRIVE, some institutions had encountered relationship challenges in their past work together, and had identified that committing to a principles-based approach in their working relationships would be helpful for this project
Shared vision	Co-creation of a common aim builds shared commitment to the partnership, and strengthens joint understanding of why partners are working together
Individual objectives	Ensures each partner has a safe space to articulate why they have joined the partnership, and what they need to get out of the partnership in order to stay engaged
	Reduces mistrust and perceptions of “hidden agendas” by encouraging openness around each partner’s individual motivations and needs
Governance	Promotes equity, ownership and transparency through codesign of decision-making structures, setting out horizontal (rather than vertical) accountability between partners
Resources for the partnership	Provides space for each partner to articulate in-kind resources (for example, expertise, buildings, information technology [IT] communications, relationships, networks) that they can bring into the partnership
	Helps to leverage the full suite of resources that each partner can tap into to advance partnership activities. This can be used to build a partnership’s work plan, and also builds equity between partners by valuing in-kind contributions
Shared and individual risks	Enables partners to identify risks that are shared across the partnership, as well as risks that are specific to individual partners
	In acknowledging that some partners have more to lose in a partnership, partners can explore ways to mitigate these risks and reduce power imbalances
Internal and external communication	Enables partners to codesign appropriate internal communication mechanisms to keep partners equitably connected across distance (e.g. remote working, poor IT connectivity) and cultures (e.g. different preferences for verbal vs written communication)
	Ensures partners have a shared understanding of how and when they will communicate about their work publicly
Intellectual property	Ensures partners have a shared understanding of how information and data outputs will be owned and shared (especially important in implementation research partnerships, where there may be divergent views around national/country data ownership)
Grievances and conflict resolution	Allows partners to jointly construct their preferred way of addressing tension or conflicts in their relationships (well in advance of a conflict arising)
Partnership review processes	Promotes mutual accountability by providing space for partners to review the effectiveness of their partnership and relationships with each other. This is “by the partners, for the partners”, rather than an external evaluation
Managing arrivals (new partners) and departures	Allows partners to jointly construct how new partners might be brought into the partnership, and how to manage the exit process should an existing partner wish to leave the partnership
Sustainability and “moving on”	Enables partners to consider ways to institutionalize partnership activities (within their own organization or by scoping an alternative source of support) beyond the lifespan of the project, or have early conversations around how to approach project completion and moving on

### Early outcomes

The process of developing the STRIVE PNG Partnership Agreement has enabled the project to extend the partnership-based approach and co-development of implementation research beyond national-level government systems and to strengthen surveillance and health systems at provincial levels. Sentinel site surveillance was first established by the PNGIMR in four key strategic locations. Through the STRIVE PNG project, PNGIMR has further established four sentinel sites in Morobe, West Sepik, Western Province and the National Capital District (NCD). By adopting the partnership-based way of working with key provincial governments and stakeholders, genuine engagement with new partners enabled fit-for-purpose implementation at each site and a demand for generating, accessing and using data in real time to make evidence-informed decisions. The partnership agreement provided a foundation to discuss key objectives and guiding principles, and to emphasize the approach the project would adopt. Through this way of working, MOUs have been signed in each province between the provincial health authorities (PHA) and PNGIMR to formalize the collaboration. Informal working groups comprising key provincial and district stakeholders from the PHA, district health offices and facility-based health staff (research nurses and officers/sisters in charge) have been established to promote ownership of data and locally led decision-making. The approach has enabled conversations about sustainability and planning for potential integration of STRIVE roles into PHA structures.

STRIVE’s internal broker uses the analogy of a “person’s home” when engaging with provincial stakeholders. The home symbolizes a province, and visitors that visit the home symbolize partners (in this case, STRIVE PNG Research Team are the visitors to the province).

*When we enter the province, we do not just “go in” and start implementing project activities, even if ethics approval has been obtained, as there are processes in place and key stakeholders that the project has to meet with who ensure that the appropriate guidance is provided for project activities to be implemented. It is the same when you are a visitor to a new home, you do not just go in and start helping yourself to food; this can be perceived as disrespectful. As a visitor, allowing that room for the host or homeowners to take lead in serving you is an act of respect towards the people and home and builds trust that eventually acts as a form of permission to work within the province. This is utilizing the project’s guiding principles which are already embedded within PNG culture.* —STRIVE PNG Internal Broker

## Example 3: partnership health check

**Table:** 

Stage	Process/how (tool)
Measuring results Reviewing efficiency and value Deepening engagement Revisiting and revising Scaling and increasing impact Moving on	Partnership health check interviews and report
	Whole of partnership meeting (conversation)

Providing opportunities for routine, periodic review of the way partners are working together is an important way to maximize a partnership’s value and embed an ongoing learning culture within a partnership. A partnership health check (PHC) is a facilitated learning process which enables partners to jointly review their collaboration practice, consider what worked well and what might be improved, identify changes in circumstances and adapt their partnership model as needed. This type of review is “by the partners, for the partners”, and not an external judgement of capacity or achievement. At the halfway mark (2020), the STRIVE PNG partners determined to jointly undertake a PHC, focusing on the following key areas as outlined in Table 3:

**Table 3:** PHC key areas and focus questions

Focus area	Focus questions
Partner roles and membership	Are all partners contributing from their place of strength (are we unlocking in-kind resources)?
	Have we got the right partners sitting at the table? Is someone missing?
Results and value-add	Have we achieved our shared objectives?
	Have your organization’s individual objectives been achieved?
	What has been the standout success for the partnership? For your organization?
Operating context	In what ways has our operating environment changed?
	How has STRIVE’s way of working influenced the partnership’s (and your organization’s) capacity to respond to these risks?
Future/next steps	What actions will we take as a result of this review?
	What have we learned that we can share with or beyond our organization?

STRIVE PNG’s Partnership Management Unit facilitated the PHC and undertook interviews with all partners (through individual and small group conversations) via Zoom, with conversations recorded for transcription purposes. Based on interview responses, the brokers developed a coding framework to guide analysis and write-up into a health check report. The report summarizes the experiences and learnings of the programme directors, expert advisors, technical leads and research officers engaged in the partnership over the first 18 months against the key focus areas. Both shared views and points of difference were acknowledged, and recommendations for future action included in the report.

In parallel with report drafting, internal brokers advanced actions to address urgent issues raised during the PHC through follow-up conversations with partners. The Partnership Management Unit designed a collaborative process for partners to review the draft report, provide feedback on key themes identified in the report via a shared visual platform (Miro board), and come together in series of whole-of-partnership meetings to discuss these key themes:

- Meeting 1: looking back—where have we come from, how are we working together, what did we learn along the way?
- Meeting 2: looking forward—how can we share resources, what can we put in place to sustain outcomes?

### Early outcomes^2^

The PHC process provided an opportunity for partners to share (through one-to-one interviews with internal brokers) honest views on both successes and challenges encountered within the partnership. It is unlikely that these perspectives would have been shared without having the “safe space” to do so via the PHC. For example, the PHC process uncovered an underlying issue not previously discussed between partners—a sense of the partnership comprising two groups: “core” partners who actively and regularly support the project’s operations and activities, and those “on the periphery” who provide technical guidance and oversight but who are not involved on a day-to-day basis. Core partners commonly felt they were contributing from their place of strength, creating a culture of ownership, while those outside the group felt disconnected from the work. This sense of inclusion and exclusion is, in fact, a common challenge faced by most partnerships during their lifespan. In STRIVE PNG’s case, it was a signal for the Partnership Management Unit to invite organizations to revisit the “partnering continuum”, and review what fit-for-purpose engagement looked like in the context of what they were trying to achieve.

For STRIVE PNG, this learning identified the need for the brokering team to support a whole-of-partnership conversation (meetings 1 and 2) to review how organizations might contribute more equitably to decision-making, and mobilize technical expertise, research support and resources within their institutions as yet untapped by the project. Importantly, this conversation also yielded clarity that some institutions did not need (or want) to be involved heavily in collaboration (joint work-planning, shared decision-making) across the partnership, as their role was focused on providing technical expertise to other partners when called upon. The PHC process, by providing space for open conversations around fit-for-purpose engagement, enabled the Partnership Management Unit to adjust project governance, work-planning and communication processes in line with technical and operational priorities, as well as what each organization felt was needed to achieve the partnership’s overall objectives.

*We have now restructured our monthly working group calls to promote the voice of research officers alongside more senior personnel (to help bridge strategic and operational thinking), agreed to rotating chairing responsibilities (by the neutral partnership broker) and introduced new communication platforms to enable “informal” ways to progressing activities, e.g. Slack & WhatsApp.* —STRIVE Internal Broker

## Conclusion

The STRIVE PNG case study describes how a partnership-based approach has been used to establish, maintain and review effective and equitable relationships between policy-makers, researchers and implementers to strengthen surveillance and health systems research in PNG. It demonstrates how processes and tools used by partnership brokers at key stages of the partnering cycle within an implementation research partnership can overcome the limitations associated with traditional research grant models which (unintentionally) entrench Global North and Global South power imbalances. Key strengths of the partnership-based approach include alignment with cultural values by paying attention to principles-based relationships, the use of co-created partnership agreements as a foundation for existing and new partnerships, and creation of an ongoing learning and reflection culture around fit-for-purpose engagement. Well-brokered research implementation partnerships lead to strengthened equity, ownership and sustainability of outcomes, and STRIVE PNG’s experience will be of interest to other collaboration groups involved in translational research for systems change.

## Data Availability

The data sets generated during and/or analysed during the current study are available from the following links.

## Abbreviations

COVID-19: Coronavirus disease 2019
STRIVE: Stronger surveillance and systems support for rapidly identifying and containing resurgent or resistant vector-borne pathogens
PHA: Provincial Health Authority
PNG: Papua New Guinea
DFAT: Department of Foreign Affairs and Trade
PNGIMR: Papua New Guinea Institute of Medical Research

## Footnotes

1 The Partnership Brokers Association is the international professional body and global standard-setter for those managing and developing collaboration processes. PBA delivers vocational training and issues formal accreditation to those who reach the required standard.

2 Outcomes shared here are key observations regarding changes to the partnership achieved as a result of conducting the process of the PHC, rather than key findings from the PHC report (which is an internal document).
